# BUILDING MORE AGE-INCLUSIVE CAMPUSES BY ENGAGING WITH RETIRED FACULTY

**DOI:** 10.1093/geroni/igae098.0986

**Published:** 2024-12-31

**Authors:** Andrea June, Caroline Cicero

**Affiliations:** Chair; Discussant

## Abstract

Identified in the Age-Friendly University (AFU) principles and in the Age Inclusivity Domains in Higher Education model, initiatives that support the campus retired and emeriti community are important elements to promoting age inclusivity and intergenerational connection. Such initiatives may be found in a myriad of forms and may include campus-specific efforts as well as organizational efforts. This symposium features campus leaders representing institutional partners of the Age-Friendly University global network who will discuss their recent efforts to understand the needs of and to engage with retired and emeriti faculty. June, Andreoletti, and Swanson (Central Connecticut State University) describe findings from an initial survey of retired and emeriti faculty that sought to understand their desire to remain engaged in intergenerational teaching, research, and service at the university. Similarly, Porter (University of Manitoba) and colleagues conducted an exploratory study at their university regarding retired academics who have specific positions as Senior Scholars and Professors Emeriti, with the goal to enhance their experiences and overall benefits for the university. Gautam (UMass Lowell) discuss their campus journey in engaging emeriti professors over the past several years and present student feedback from an “intergenerational team talk” activity which engages a retired professor and students in a Gerontology course. Finally, Montepare (Lasell University) reports on data collected from the American Psychological Association’s late career stage/retired faculty and addresses how professional organizations could support age-inclusive campus practices with more age-inclusive member practices. Cicero (University of Southern California) will share insights and pose future directions as the discussant.

